# A Splenic Infarct in Scrub Typhus: A Rare Entity

**DOI:** 10.7759/cureus.64261

**Published:** 2024-07-10

**Authors:** Sonali Ghosh, Atish Akhuli, Sourav Das Choudhury, Kaushik Ghosh

**Affiliations:** 1 Emergency Medicine and Critical Care, Institute of Post-Graduate Medical Education and Research and Seth Sukhlal Karnani Memorial Hospital, Kolkata, IND; 2 Anaesthesiology, Murshidabad Medical College and Hospital, Berhampore, IND; 3 Internal Medicine, Mediflora, Berhampore, IND; 4 Medicine, KPC Medical College and Hospital, Kolkata, IND; 5 Medicine, Murshidabad Medical College and Hospital, Berhampore, IND

**Keywords:** computed tomography abdomen, doxycycline, orientia tsutsugamushi, splenic infarct, scrub typhus

## Abstract

Scrub typhus is a zoonotic feverish condition that can range from mild to severe, potentially life-threatening symptoms. Common signs include fever, headache, muscle pain, and a skin rash. Although rare, splenic infarction is a known complication of scrub typhus, with only a limited number of cases documented in medical literature. The case of a 68-year-old male with fever and abdominal discomfort, ultimately diagnosed with both scrub typhus and splenic infarct, illustrates the importance of recognizing splenic infarction as a potential complication of scrub typhus, particularly in areas where the disease is prevalent. The patient was promptly diagnosed and managed with a favorable outcome.

## Introduction

Scrub typhus, also known as tsutsugamushi disease, is a rickettsial infection caused by gram-negative *Orientia tsutsugamushi*. It is transmitted to humans through the bite of infected larval mites, known as chiggers. These mites are commonly found in rural areas of endemic regions, such as Southeast Asia, the Western Pacific Islands, and parts of South Asia [1]. In endemic regions, outbreaks of scrub typhus can occur during the rainy season, when chiggers are more active. Clinical features of scrub typhus can vary widely, but most commonly include fever, headache, myalgia, and rash. Other symptoms may include coughing, lymphadenopathy, and gastrointestinal symptoms. If left untreated, scrub typhus can lead to severe complications such as pneumonia, acute respiratory distress syndrome (ARDS), acute kidney injury, hepatitis, gastrointestinal bleeding, myocarditis with shock, and central nervous system involvement, including multiorgan failure [2]. Scrub typhus most commonly involves the intraabdominal organs, the common ones being the liver, gall bladder, kidneys, gastrointestinal tract, and pancreas. However, clinical involvement of the spleen is rarely reported. We report a case of scrub typhus infection manifesting with a splenic infarct in a tertiary care hospital in eastern India.

## Case presentation

A 68-year-old male without any underlying health conditions presented with a month-long history of high-grade intermittent fever accompanied by chills and rigors. The patient also complained of persistent dull, aching pain in the upper left abdomen, which initially manifested suddenly with colicky characteristics. Although the fever had decreased in intensity over the past two days, the abdominal pain persisted. Despite treatment with antispasmodics, the pain remained, and the patient experienced two instances of non-projectile vomiting. Physical examination revealed signs of dehydration and generalized emaciation. He was mildly tachypneic, with a respiratory rate of 22 breaths per minute and oxygen saturation of 94% on room air. Mild lower lobe crepitations were detected during respiratory system assessment, while other systems remained largely unremarkable.

**Figure 1:**
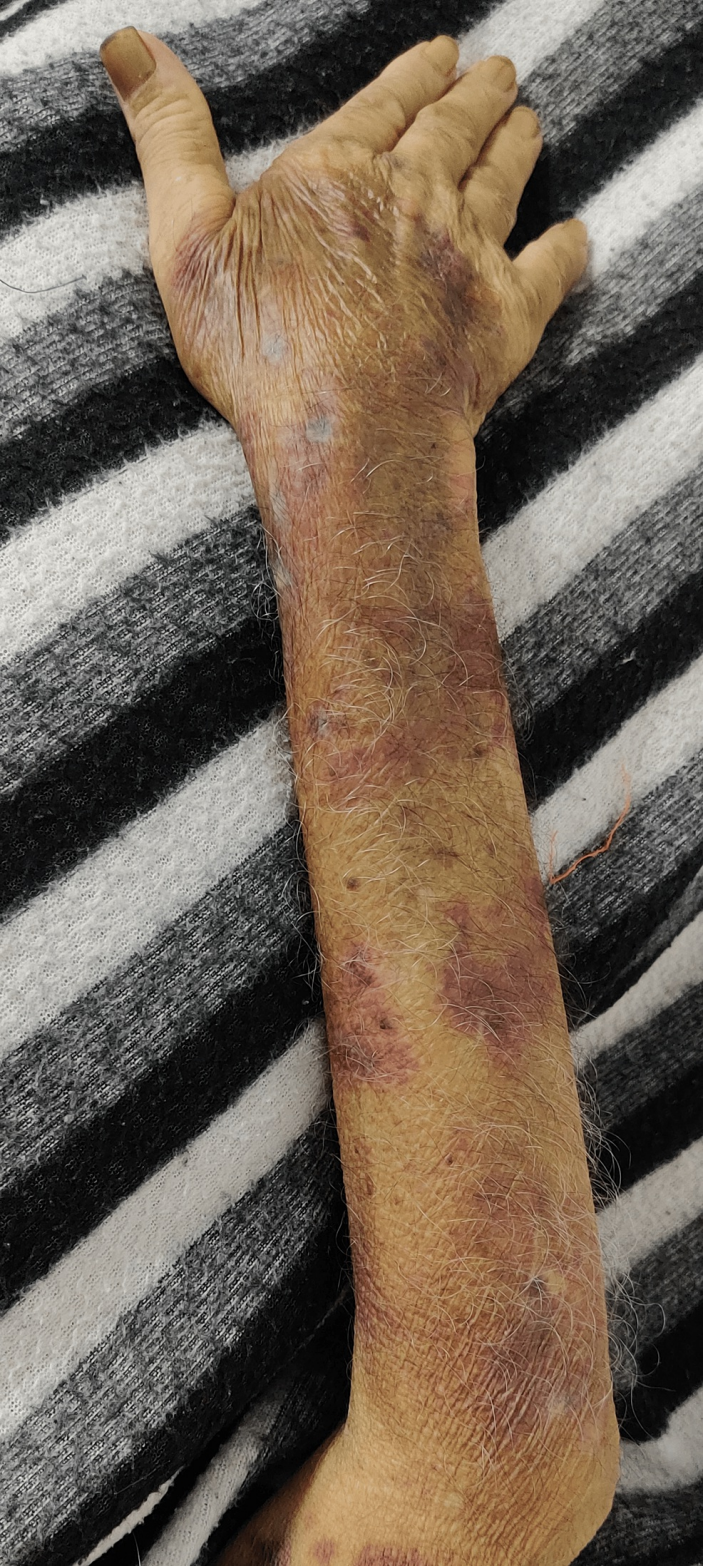
The patient exhibited ecchymosis throughout his body without any signs of eschar.

Mild pallor was observed, with no signs of lymphadenopathy. Mild tenderness was noted in the left upper abdominal quadrant, along with a mildly palpable spleen. The patient exhibited ecchymosis throughout his body but no signs of eschar (Figure 1).

The results of the complete blood count indicated a low hemoglobin level of 9.5 g/dl, a normal leucocyte count of 4000/cumm with a distribution of neutrophils (77%), lymphocytes (17%), monocytes (4%), eosinophils (2%), and a platelet count of 1.01 x 103/mcl. The liver function test revealed decreased protein levels (total protein: 5.6 g/dl, albumin: 2.9 g/dl) and a slight elevation in liver enzymes (serum glutamic pyruvic transaminase (SGPT): 77.2 IU/L, serum glutamic oxaloacetic transaminase (SGOT): 43.2 IU/L, and alkaline phosphatase: 350 IU/L). Additionally, the levels of urea (27 mg/dl), creatinine (0.6 mg/dl), sodium (141 mmol/L), and potassium (4 mmol/L) were all within the normal range. His serum amylase and lipase levels were also within the normal range. Pancreatitis was ruled out by serum amylase and lipase levels, along with radiological features.

Infective etiologies of fever were evaluated. A peripheral smear for malaria parasites was negative. Dengue fever and enteric fever were ruled out as negative by appropriate serological tests. The diagnosis of scrub typhus was confirmed by a strongly positive IgM enzyme-linked immunosorbent assay.

**Figure 2:**
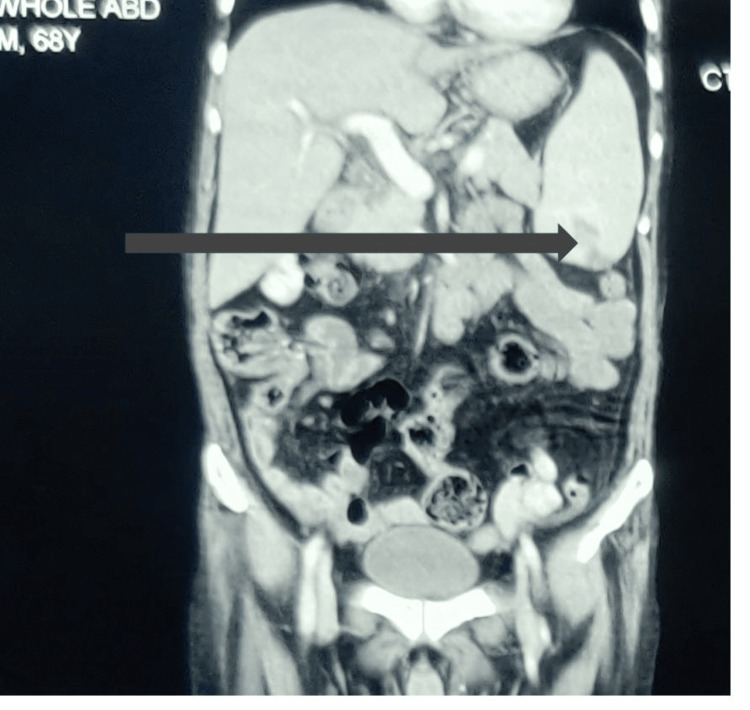
Contrast-enhanced computed tomography shows the presence of a hypodense wedge-shaped area without any contrast enhancement at the inferolateral pole of the spleen.

Ultrasound showed an enlarged spleen and a hypoechoic, well-defined wedge-shaped lesion in the lower pole of the spleen, suggestive of a splenic infarct. Other organs were normal except for the presence of a contracted and thickened gallbladder. Contrast-enhanced computed tomography (CECT) confirmed the presence of a hypodense wedge-shaped area without any contrast enhancement at the inferolateral pole of the spleen, sparing the subcapsular space and bilateral pleural effusion (Figure 2).

The patient's initial treatment included administering intravenous fluids, broad-spectrum antibiotics, antipyretics, and other supportive therapies. Upon receiving the scrub typhus reports, intravenous doxycycline (100 mg) twice daily was initiated. By the third day of treatment, the patient was afebrile with a notable reduction in abdominal pain. The patient was discharged on the fifth day of hospitalization with stable vital signs.

## Discussion

Scrub typhus is a bacterial infection caused by *Orientia tsutsugamushi* that is transmitted to humans through the bite of infected chiggers. This infection is commonly found in rural areas of Asia, including countries like India, China, Japan, and South Korea [1]. The clinical presentation of scrub typhus can vary from mild symptoms to severe complications, depending on the individual’s immune response and the strain of bacteria.

One of the potential complications of scrub typhus is a splenic infarct [3,4,5]. Splenic infarction occurs when the blood supply to the spleen is compromised, leading to tissue damage and necrosis. This can result in symptoms such as left upper abdominal pain, fever, and sometimes an enlarged spleen. However, the incidence of splenic infarction is extremely rare. To the best of our knowledge, only nine cases have been reported so far.

The mechanism by which scrub typhus can lead to splenic infarction is not entirely clear, but it is believed to be related to the systemic inflammatory response triggered by the infection [5, 6]. The bacteria can cause endothelial cell damage, leading to microvascular thrombosis and ischemia in various organs, including the spleen.

Diagnosis of splenic infarction in scrub typhus typically involves imaging studies such as CT scans or ultrasounds, which can show characteristic findings of infarction in the spleen.

The existing treatment approach for scrub typhus involves administering antibiotics such as doxycycline or azithromycin. In cases of mild-to-moderate infection where doxycycline is the chosen antibiotic, the recommended treatment duration ranges from one to two weeks.

Treatment usually involves supportive care, including pain management and monitoring for complications such as splenic abscess and rupture of the spleen [7]. In severe cases, surgical intervention may be necessary [7].

## Conclusions

The development of a splenic infarct in scrub typhus highlights the importance of early recognition and appropriate management of this potentially serious infection. Close monitoring and prompt treatment can help prevent complications and improve outcomes for patients with scrub typhus. In the endemic zone, it is important to investigate potential causes of splenic infarctions, as early identification can facilitate prompt initiation of appropriate treatment.
